# Supplementary material for: Knowledge of Maternal Health Complications: A Critical Analysis Among Pregnant Women in Bangladesh
Source: PLOS Glob Public Health. 2025 Nov 13;5(11):e0005469. doi: 10.1371/journal.pgph.0005469 (PMC12614543; doi:10.1371/journal.pgph.0005469)
Supplement: S1 Table — (DOCX) [file pgph.0005469.s001.docx]

**S1 Table: Bivariate analysis of knowledge of specific maternal health complications by sociodemographic characteristics among currently pregnant women aged 15 – 49 years in Bangladesh, 2016 BMMS [N =5,625]**

| **Variables** | **Severe headache** | | **Blurred vision** | | **High blood pressure** | | **Edema / Pre-Eclampsia** | | **Convulsion /Eclampsia / Unconsciousness** | | **Excess vaginal bleeding** | | **Foul-smelling discharge with severe fever** | | **Jaundice** | |
| --- | --- | --- | --- | --- | --- | --- | --- | --- | --- | --- | --- | --- | --- | --- | --- | --- |
|  | **Yes** | **No** | **Yes** | **No** | **Yes** | **No** | **Yes** | **No** | **Yes** | **No** | **Yes** | **No** | **Yes** | **No** | **Yes** | **No** |
| **Gestational Age** | | | | | | | | | | | | | | | | |
| 1^st^ Trimester | 271 (18.1) | 1226 (81.9) | 165  (11.0) | 1332 (89.0) | 249 (16.6) | 1248 (83.4) | 279 (18.6) | 1219 (81.4) | 538 (36.0) | 959  (64.0) | 290 (19.4) | 1208 (80.6) | 40 (2.7) | 1458 (97.3) | 122 (8.1) | 1376 (91.9) |
| 2^nd^ Trimester | 435 (19.3) | 1819 (80.7) | 224 (9.9) | 2030 (90.1) | 414 (18.4) | 1840 (81.6) | 396 (17.6) | 1858 (82.4) | 852 (37.8) | 1402 (62.2) | 385 (17.1) | 1870 (82.9) | 63 (2.8) | 2192 (97.2) | 175 (7.7) | 2080 (92.3) |
| 3^rd^ Trimester | 350  (18.7) | 1524  (81.3) | 225  (12.0) | 1649  (88.0) | 418  (22.3) | 1455  (77.7) | 333  (17.8) | 1541  (82.2) | 654  (34.9) | 1220  (65.1) | 302  (16.1) | 1571  (83.9) | 45  (2.4) | 1829  (97.6) | 155  (8.3) | 1719  (91.7) |
| *p -* value | 0.735 | | 0.201 | | 0.001 | | 0.769 | | 0.229 | | 0.104 | | 0.798 | | 0.859 | |
| **Birth order** | | | | | | | | | | | | | | | | |
| 0 | 448 (19.4) | 1,868 (80.6) | 264 (11.4) | 2,052 (88.6) | 445 (19.2) | 1,871 (80.8) | 416 (17.9) | 1,900 (82.1) | 727 (31.4) | 1,589 (68.6) | 340 (14.7) | 1,976 (85.3) | 55 (2.4) | 2,261 (97.6) | 201 (8.7) | 2,115 (91.3) |
| 1 | 352 (19.3) | 1,471 (80.7) | 195 (10.7) | 1,628 (89.3) | 382 (21.0) | 1,441 (79.0) | 313 (17.2) | 1,510 (82.8) | 773 (42.4) | 1,050 (57.6) | 353 (19.4) | 1,470 (80.6) | 49 (2.7) | 1,774 (97.3) | 127 (6.9) | 1,696 (93.1) |
| 2 – 3 | 220 (17.4) | 1,044 (82.6) | 129 (10.2) | 1,135 (89.8) | 216 (17.1) | 1,048 (82.9) | 239 (18.9) | 1,026 (81.1) | 468 (37.0) | 796  (63.0) | 239 (18.9) | 1,025 (81.1) | 40 (3.2) | 1,224 (96.8) | 103 (8.2) | 1,161 (91.8) |
| 4 + | 35 (15.8) | 187 (84.2) | 27 (12.0) | 195 (88.0) | 37 (16.8) | 185 (83.2) | 40 (18.0) | 182 (82.0) | 76 (34.1) | 146  (65.9) | 44 (19.8) | 178 (80.2) | 3 (1.1) | 219 (98.9) | 20 (9.2) | 202 (90.8) |
| *p -* value | 0.428 | | 0.779 | | 0.117 | | 0.764 | | <0.001 | | 0.002 | | 0.369 | | 0.325 | |
| **Age (years)** | | | | | | | | | | | | | | | | |
| <20 | 270 (16.2) | 1,392 (83.8) | 186 (11.2) | 1476 (88.8) | 251 (15.1) | 1,411 (84.9) | 280 (16.8) | 1,382 (83.2) | 464 (27.9) | 1,198 (72.1) | 214 (12.9) | 1,448 (87.1) | 37 (2.2) | 1,625 (97.8) | 134 (8.1) | 1,528 (91.9) |
| 20 – 24 | 387 (20.2) | 1,533 (79.8) | 211 (11.0) | 1710 (89.0) | 407 (21.2) | 1,513 (78.8) | 345 (18.0) | 1,576 (82.0) | 726 (37.8) | 1,194 (62.2) | 338 (17.6) | 1,582 (82.4) | 47 (2.5) | 1,873 (97.5) | 156 (8.1) | 1,764 (91.9) |
| 25 – 29 | 259 (19.9) | 1,045 (80.1) | 135 (10.3) | 1169 (89.7) | 275 (21.0) | 1,030 (79.0) | 224 (17.2) | 1,080 (82.8) | 567 (43.5) | 737  (56.5) | 288 (22.1) | 1,017 (77.9) | 36 (2.8) | 1,268 (97.2) | 103 (7.9) | 1,201 (92.1) |
| 30+ | 140 (18.9) | 599 (81.1) | 83 (11.2) | 656 (88.8) | 149 (20.1) | 590 (79.9) | 159 (21.5) | 580 (78.5) | 287 (38.8) | 452  (61.2) | 137 (18.5) | 602 (81.5) | 27 (3.6) | 712 (96.4) | 58 (7.8) | 681 (92.2) |
| *p -* value | 0.059 | | 0.922 | | <0.001 | | 0.119 | | <0.001 | | <0.001 | | 0.395 | | 0.994 | |
| **Educational Attainment** | | | | | | | | | | | | | | | | |
| No education | 45 (11.5) | 343 (88.5) | 39  (9.9) | 349 (90.1) | 48 (12.3) | 340 (87.7) | 73 (18.8) | 315 (81.2) | 96 (24.8) | 291  (75.2) | 45 (11.7) | 342 (88.3) | 7 (1.7) | 381 (98.3) | 32 (8.4) | 355 (91.6) |
| Primary incomplete | 132 (16.0) | 694 (84.0) | 71  (8.6) | 755 (91.4) | 124 (15.0) | 702 (85.0) | 127 (15.4) | 699 (84.6) | 238 (28.8) | 588  (71.2) | 142 (17.2) | 684 (82.8) | 25 (3.0) | 801 (97.0) | 72 (8.7) | 754 (91.3) |
| Primary complete | 113 (14.6) | 661 (85.4) | 45  (5.8) | 730 (94.2) | 121 (15.6) | 654 (84.4) | 129 (16.7) | 645 (83.3) | 286 (36.9) | 488  (63.1) | 109 (14.1) | 666 (85.9) | 17 (2.2) | 757 (97.8) | 47 (6.0) | 728 (94.0) |
| Secondary incomplete | 447 (18.9) | 1,922 (81.1) | 292 (12.3) | 2,077 (87.7) | 445 (18.8) | 1,924 (81.2) | 393 (16.6) | 1,976 (83.4) | 846 (35.7) | 1,523 (64.3) | 400 (16.9) | 1,969 (83.1) | 53 (2.3) | 2,315 (97.7) | 188 (7.9) | 2,181 (92.1) |
| Secondary complete or higher | 319 (25.2) | 949 (74.8) | 169 (13.3) | 1,100 986.7) | 344 (27.1) | 924 (72.9) | 286 (22.6) | 982 (77.4) | 578 (45.6) | 690  (54.4) | 280 (22.1) | 988 (77.9) | 45 (3.5) | 1,224 (96.5) | 112 (8.8) | 1,156 (91.2) |
| *p -* value | <0.001 | | <0.001 | | <0.001 | | 0.001 | | <0.001 | | <0.001 | | 0.217 | | 0.382 | |
| **Access to Any Media (Newspaper, Radio, TV)** | | | | | | | | | | | | | | | | |
| At least once a week | 675  (20.6) | 2,608  (79.4) | 396 (12.0) | 2,888  (88.0) | 734 (22.3) | 347 (14.8) | 606 (18.4) | 402 (17.2) | 1,261 (38.4) | 784 (33.5) | 633 (19.3) | 2,651 (80.7) | 95 (2.9) | 3,189 (97.1) | 279 (8.5) | 3,005 (91.5) |
| Less than once a week | 381  (16.3) | 1,961  (83.7) | 219  (9.3) | 2,123 (90.7) | 2,550 (77.7) | 1,995 (85.2) | 2,678 (81.6) | 1,940 (82.8) | 2,023 (61.6) | 1,558 (66.5) | 344 (14.7) | 1,998  (85.3) | 52 (2.2) | 2,289 (97.8) | 172 (7.4) | 2,169 (92.6) |
| *p -* value | 0.001 | | 0.004 | | <0.001 | | 0.298 | | 0.002 | | <0.001 | | 0.196 | | 0.192 | |
| **Wealth quintile** | | | | | | | | | | | | | | | | |
| Lowest | 199 (17.3) | 951 (82.7) | 119 (10.3) | 1,031 (89.7) | 163 (14.2) | 987 (85.8) | 197 (16.9) | 955 (83.1) | 368 (32.0) | 782  (68.0) | 169 (14.7) | 981 (85.3) | 18 (1.6) | 1,131 (98.4) | 86 (92.5) | 1,064 (92.5) |
| Second | 173 (15.4) | 954 (84.6) | 95  (8.4) | 1,033 (91.6) | 189 (16.7) | 939 (83.3) | 167 (14.8) | 961 (85.2) | 391 (34.7) | 736  (65.3) | 173 (15.4) | 954 (84.6) | 30 (2.6) | 1,098 (97.4) | 88 (7.8) | 1,039 (92.2) |
| Middle | 204 (19.3) | 857 (80.7) | 123 (11.6) | 939 (88.4) | 203 (19.1) | 859 (80.9) | 188 (17.7) | 873 (82.3) | 370 (34.9) | 691  (65.1) | 195 (18.3) | 867 (81.7) | 32 (3.0) | 1,030 (97.0) | 102 (9.6) | 960 (90.4) |
| Fourth | 233 (18.9) | 998 (81.1) | 132 (10.7) | 1,099 (89.3) | 255 (20.7) | 976 (79.3) | 212 (17.2) | 1,019 (82.8) | 436 (35.5) | 794  (64.5) | 208 (16.9) | 1,023 (83.1) | 32 (2.6) | 1,199 (97.4) | 100 (8.1) | 1,131 (91.9) |
| Highest | 247 (23.4) | 857 (80.7) | 146 (13.9) | 910 (86.1) | 272 (25.7) | 784 (74.3) | 247 (23.4) | 809 (76.6) | 479 (45.3) | 577  (54.7) | 232 (22.0) | 824 (78.0) | 36 (3.4) | 1,020 (96.6) | 75 (7.1) | 981 (92.9) |
| *p -* value | 0.001 | | 0.014 | | <0.001 | | <0.001 | | <0.001 | | 0.001 | | 0.181 | | 0.493 | |
| **Place of Residence** | | | | | | | | | | | | | | | | |
| Urban | 312 (21.1) | 1,166 (78.9) | 182  (12.3) | 1,296 (87.7) | 343 (23.2) | 1,134 (76.8) | 287 (19.5) | 1,190 (80.5) | 547 (37.0) | 930  (63.0) | 264 (17.9) | 1,213 (82.1) | 45 (3.1) | 1,432 (96.9) | 126 (8.5) | 1,352 (91.5) |
| Rural | 744 (17.9) | 3,404 (82.1) | 433 (10.4) | 3715 (89.6) | 738 (17.8) | 3,410 (82.2) | 720 (17.4) | 3,428 (82.6) | 1,497 (36.1) | 2,651 (63.9) | 713 (17.2) | 3,435 (82.8) | 102 (2.5) | 4,046 (97.5) | 326 (7.8) | 3,822 (92.2) |
| *p -* value | 0.022 | | 0.089 | | <0.001 | | 0.122 | | 0.635 | | 0.623 | | 0.284 | | 0.511 | |
| **Division** | | | | | | | | | | | | | | | | |
| Barisal | 86 (28.8) | 214 (71.2) | 33 (11.0) | 267 (89.0) | 78 (26.0) | 222 (74.0) | 61 (20.4) | 239 (79.6) | 127 (42.3) | 173  (57.7) | 25.9 (8.6) | 274 (91.4) | 8 (2.6) | 292 (97.0) | 21 (7.0) | 279 (93.0) |
| Chittagong | 190 (14.1) | 1161 (85.9) | 94  (7.0) | 1,256 (93.0) | 211 (15.6) | 1,139 (84.4) | 192 (14.2) | 1,159 (85.8) | 379 (42.3) | 972  (71.9) | 252 (18.7) | 1,098 (81.3) | 32 (2.3) | 1,319 (97.7) | 124 (9.2) | 1,227 (90.8) |
| Dhaka | 306 (20.9) | 1,155 (79.1) | 209 (14.3) | 1,252 (85.7) | 324 (22.2) | 1,136 (77.8) | 298 (20.4) | 1,163 (79.6) | 573 (39.2) | 888  (60.8) | 293 (20.1) | 1,167 (79.9) | 55 (3.8) | 1,405 (96.2) | 113 (7.8) | 1,347 (92.2) |
| Khulna | 124 (23.1) | 411 (76.9) | 78 (14.5) | 457 (85.5) | 115 (21.4) | 420 (78.6) | 124 (23.2) | 411 (76.8) | 221 (41.4) | 313  (58.6) | 81 (15.2) | 454 (84.8) | 14 (2.6) | 521 (97.4) | 36.5 (6.8) | 498 (93.2) |
| Mymensingh | 47 (10.3) | 413 (89.7) | 23  (5.0) | 437 (95.0) | 71 (15.5) | 389 (84.5) | 54 (11.8) | 406 (88.2) | 207 (44.9) | 253  (55.1) | 100 (21.7) | 360 (78.3) | 11 (2.3) | 449 (97.7) | 23 (5.0) | 437 (95.0) |
| Rajshahi | 139 (23.8) | 445 (76.2) | 83 (14.2) | 501 (85.8) | 130 (22.2) | 455 (77.8) | 129 (22.1) | 455 (77.9) | 188 (32.1) | 397  (67.9) | 90 (15.4) | 494 (84.6) | 6 (1.0) | 578 (99.0) | 53 (10.0) | 531 (90.9) |
| Rangpur | 99 (18.6) | 436 (81.4) | 58 (10.8) | 478 (89.2) | 96 (17.9) | 440 (82.1) | 87 (16.3) | 448 (83.7) | 226 (42.1) | 310  (57.9) | 77 (14.3) | 459 (85.7) | 15 (2.7) | 521 (97.3) | 54 (10.0) | 482 (90.0) |
| Sylhet | 65 (16.2) | 335 (83.8) | 37  (9.3) | 362 (99.7) | 56 (14.1) | 343 (85.9) | 62.5 (15.6) | 337 (84.4) | 125 (31.2) | 274  (68.8) | 58 (14.4) | 342 (85.6) | 7 (1.0) | 392 (98.2) | 27 (6.7) | 372 (93.3) |
| *p -* value | <0.001 | | <0.001 | | <0.001 | | <0.001 | | <0.001 | | <0.001 | | 0.050 | | 0.116 | |

**S1 Table (continued)**

| **Variables** | **Tetanus** | | **Mal-presentation** | | **Prolonged labor** | | **Obstructed labor** | | **Delayed cord presentation** | | **Ruptured membrane** | | **Other** | | **Don’t know** | |
| --- | --- | --- | --- | --- | --- | --- | --- | --- | --- | --- | --- | --- | --- | --- | --- | --- |
|  | **Yes** | **No** | **Yes** | **No** | **Yes** | **No** | **Yes** | **No** | **Yes** | **No** | **Yes** | **No** | **Yes** | **No** | **Yes** | **No** |
| **Gestational Age** | | | | | | | | | | | | | | | | |
| 1^st^ Trimester | 241 (16.1) | 1256 (83.9) | 235  (15.7) | 1263 (84.3) | 229 (15.3) | 1268 (84.7) | 111 (7.4) | 1387 (92.6) | 378  (25.2) | 1120  (74.8) | 155  (10.4) | 1342 (89.6) | 32  (2.2) | 1465 (97.8) | 236 (15.7) | 1262 (84.3) |
| 2^nd^ Trimester | 444 (19.7) | 1810 (80.3) | 362 (16.1) | 1892 (83.9) | 400 (17.7) | 1855 (82.3) | 146 (6.5) | 2108 (93.5) | 598  (26.5) | 1657 (73.5) | 240  (10.7) | 2014 (89.3) | 63  (2.8) | 2191 (97.2) | 347 (15.4) | 1907 (84.6) |
| 3^rd^ Trimester | 292  (15.6) | 1582  (84.4) | 352  (18.8) | 1521  (81.2) | 264  (14.1) | 1609  (85.9) | 95  (5.1) | 1778  (94.9) | 491  (26.2) | 1383  (73.8) | 215  (11.5) | 1658  (88.5) | 53  (2.8) | 1821  (97.2) | 274  (14.6) | 1600  (85.4) |
| *p -* value | 0.005 | | 0.076 | | 0.027 | | 0.053 | | 0.763 | | 0.643 | | 0.571 | | 0.709 | |
| **Birth order** | | | | | | | | | | | | | | | | |
| 0 | 397 (17.2) | 1,919 (82.8) | 370 (16.0) | 1,946 (82.7) | 332 (14.3) | 1,987 (85.7) | 127 (5.5) | 2,189 (94.5) | 540 (23.3) | 1,776 (76.7) | 213 (9.2) | 2,103 (90.8) | 55  (2.4) | 2,261 (97.6) | 434 (18.8) | 1,882 (81.2) |
| 1 | 319 (17.5) | 1,504 (82.5) | 316 (17.3) | 1,508 (82.7) | 322 (17.7) | 1,501 (82.3) | 116 (6.4) | 1,707 (93.6) | 501 (27.5) | 1,322 (72.5) | 202 (11.1) | 1,621 (88.9) | 52  (2.9) | 1,771 (97.1) | 246 (13.5) | 1,578 (86.5) |
| 2 – 3 | 220 (17.4) | 1,045 (82.6) | 227 (17.9) | 1,037 (82.1) | 206 (16.3) | 1,058 (83.7) | 91  (7.2) | 1,173 (92.8) | 360 (28.5) | 904 (71.5) | 153 (12.1) | 1,111 (87.9) | 34  (2.7) | 1,230 (97.3) | 151 (12.0) | 1,113 (88.0) |
| 4 + | 41 (18.5) | 181 (81.5) | 37 (16.6) | 185 (83.4) | 33 (14.9) | 189 (85.1) | 18  (8.3) | 203 (91.7) | 64 (28.8) | 158 (71.2) | 42 (19.0) | 180 (81.0) | 7  (3.0) | 215 (97.0) | 26 (11.5) | 196 (88.5) |
| *p -* value | 0.974 | | 0.590 | | 0.078 | | 0.194 | | 0.009 | | <0.001 | | 0.856 | | <0.001 | |
| **Age (years)** | | | | | | | | | | | | | | | | |
| <20 | 255 (15.4) | 1,407 (84.6) | 261 (15.7) | 1,401 (84.3) | 217 (13.1) | 1,445 (86.9) | 95  (5.7) | 1,567 (94.3) | 370 (22.3) | 1,292 (77.7) | 145 (8.7) | 1,516 (91.3) | 38  (2.3) | 1,624 (97.7) | 378 (22.8) | 1,284 (77.2) |
| 20 – 24 | 326 (17.0) | 1,595 (83.0) | 310 (16.1) | 1,611 (83.9) | 304 (15.8) | 1,616 (84.2) | 123 (6.4) | 1,797 (93.6) | 528 (27.5) | 1,393 (72.5) | 218 (11.4) | 1,702 (88.6) | 50.0 (2.6) | 1,871 (97.4) | 255 (13.3) | 1,665 (86.7) |
| 25 – 29 | 251 (19.2) | 1,054 (80.8) | 238 (18.2) | 1,066 (81.8) | 251 (19.2) | 1,053 (80.8) | 87  (6.7) | 1,217 (93.3) | 361 (27.7) | 943 (72.3) | 140 (10.8) | 1,164 (89.2) | 34  (2.6) | 1,270 (97.4) | 145 (11.1) | 1,159 (88.9) |
| 30+ | 145 (19.7) | 594 (80.3) | 141 (19.1) | 598 (80.9) | 121 (16.3) | 618 (83.7) | 47  (6.4) | 692 (93.6) | 207 (28.0) | 532 (72.0) | 107 (14.5) | 632 (85.5) | 27  (3.6) | 712 (96.4) | 78 (10.6) | 661 (89.4) |
| *p -* value | 0.056 | | 0.207 | | 0.002 | | 0.778 | | 0.006 | | 0.003 | | 0.522 | | <0.001 | |
| **Educational Attainment** | | | | | | | | | | | | | | | | |
| **No education** | 35  (9.0) | 353 (91.0) | 50 (12.8) | 338 (87.2) | 60 (15.5) | 328 (84.5) | 22  (5.7) | 366 (94.3) | 102 (26.4) | 285 (73.6) | 38  (9.7) | 350 (90.3) | 12  (3.0) | 376 (97.0) | 86 (22.1) | 302 (77.9) |
| Primary incomplete | 118 (14.3) | 708 (85.7) | 146 (17.6) | 681 (82.4) | 119 (14.4) | 707 (85.6) | 63  (7.7) | 763 (92.3) | 245 (29.7) | 581 (70.3) | 102 (12.4) | 724 (87.6) | 20  (2.4) | 807 (97.6) | 120 (14.6) | 706 (85.4) |
| Primary complete | 105 (13.5) | 670 (86.5) | 138 (17.8) | 637 (82.2) | 120 (15.5) | 655 (84.5) | 42  (5.4) | 733 (94.6) | 215 (27.8) | 559 (72.2) | 99 (12.8) | 675 (87.2) | 15  (2.0) | 759 (98.0) | 137 (17.7) | 637 (82.3) |
| Secondary incomplete | 431 (18.2) | 1,938 (81.8) | 357 (15.1) | 2,012 (84.9) | 376 (15.9) | 1,993 (84.1) | 139 (5.9) | 2,230 (94.1) | 576 (24.3) | 1,793 (75.7) | 237 (10.0) | 2,132 (90.0) | 56  (2.4) | 2,312 (97.6) | 393 (16.6) | 1,976 (83.4) |
| Secondary complete or higher | 289 (22.8) | 980 (77.2) | 259 (20.4) | 1,009 (79.6) | 218 (17.2) | 1,050 (82.8) | 86  (6.8) | 1,180 (93.2) | 327 (25.8) | 941 (74.2) | 135 (10.6) | 1,134 (89.4) | 45  (3.5) | 1,223 (96.5) | 121 (9.5) | 1,147 (90.5) |
| *p -* value | <0.001 | | 0.003 | | 0.700 | | 0.405 | | 0.133 | | 0.256 | | 0.349 | | <0.001 | |

| **Access to Any Media (Newspaper, Radio, TV)** | | | | | | | | | | | | | | | | |
| --- | --- | --- | --- | --- | --- | --- | --- | --- | --- | --- | --- | --- | --- | --- | --- | --- |
| At least once a week | 579  (17.6) | 2,704  (82.4) | 589 (17.9) | 2,694  (82.1) | 561 (17.1) | 2,723 (82.9) | 213 (6.5) | 3,070 (93.5) | 856 (26.1) | 2,427 (73.9) | 348 (10.6) | 2,936 (89.4) | 89 (2.7) | 3,195 (97.3) | 459 (14.0) | 2,824 (86.0) |
| Less than once a week | 398  (17.0) | 1,944  (83.0) | 360  (15.4) | 1,981 (84.6) | 332 (14.2) | 2,010 (85.8) | 139 (6.0) | 2,202 (94.0) | 610 (26.0) | 1,732 (74.0) | 263 (11.2) | 2,078  (88.8) | 59 (2.5) | 2,283 (97.5) | 398 (17.0) | 1,944 (83.0) |
| *p -* value | 0.5889 | | 0.036 | | 0.012 | | 0.468 | | 0.971 | | 0.493 | | 0.741 | | 0.588 | |

| **Wealth quintile** | | | | | | | | | | | | | | | | |
| --- | --- | --- | --- | --- | --- | --- | --- | --- | --- | --- | --- | --- | --- | --- | --- | --- |
| Lowest | 191 (16.6) | 959 (83.4) | 174 (15.2) | 975 (84.8) | 193 (16.8) | 956 (83.2) | 82  (7.1) | 1,068 (92.9) | 334 (29.1) | 815 (70.9) | 138 (12.0) | 1,011 (88.0) | 32  (2.8) | 1,117 (97.2) | 201 (17.5) | 948 (82.5) |
| Second | 203 (18.0) | 925 (82.0) | 164 (14.6) | 963 (85.4) | 161 (14.3) | 967 (85.7) | 73  (6.5) | 1,055 (93.5) | 291 (25.8) | 836 (74.2) | 125 (11.1) | 1,003 (88.9) | 17  (1.5) | 1,111 (98.5) | 188 (16.7) | 940 (83.3) |
| Middle | 184 (17.4) | 877 (82.6) | 201 (18.9) | 860 (81.1) | 158 (14.9) | 904 (85.1) | 60  (5.7) | 1,001 (94.3) | 265 (24.9) | 797 (75.1) | 127 (12.0) | 934 (88.0) | 34  (3.2) | 1,028 (96.8) | 160 (15.1) | 901 (84.9) |
| Fourth | 218 (17.7) | 1,013 (82.3) | 209 (17.0) | 1,022 (83.0) | 185 (15.3) | 1,042 (84.7) | 67  (5.5) | 1,163 (94.5) | 320 (26.0) | 911 (74.0) | 122 (9.9) | 1,108 (90.1) | 34  (2.7) | 1,197 (97.3) | 181 (14.7) | 1,050 (85.3) |
| Highest | 182 (17.2) | 874 (82.8) | 201 (19.0) | 856 (81.0) | 193 (18.2) | 863 (81.8) | 71  (6.7) | 985 (93.3) | 256 (24.3) | 799 (75.7) | 98  (9.3) | 958 (90.7) | 32  (3.0) | 1,024 (97.0) | 127 (12.0) | 930 (88.0) |
| *p -* value | 0.955 | | 0.052 | | 0.187 | | 0.594 | | 0.316 | | 0.303 | | 0.313 | | 0.024 | |
| **Place of Residence** | | | | | | | | | | | | | | | | |
| Urban | 227 (15.4) | 1,250 (84.6) | 261 (17.7) | 1,217 (82.3) | 231 (15.6) | 1,246 (84.4) | 87  (5.9) | 1,391 (94.1) | 353 (23.9) | 1,124 (76.1) | 155 (10.5) | 1,322 (89.5) | 38  (2.6) | 1,439 (97.4) | 230 (15.5) | 1,248 (84.5) |
| Rural | 750 (18.1) | 3,398 (81.9) | 689 (16.6) | 3,459 (83.4) | 662 (16.0) | 3,486 (84.0) | 266 (6.4) | 3,882 (93.6) | 1113 (26.8) | 3,035 (73.2) | 456 (11.0) | 3,692 (89.0) | 110 (2.7) | 4,038 (97.3) | 627 (15.1) | 3,521 (84.9) |
| *p -* value | 0.037 | | 0.429 | | 0.808 | | 0.497 | | 0.077 | | 0.656 | | 0.879 | | 0.738 | |
| **Division** | | | | | | | | | | | | | | | | |
| Barisal | 68 (22.6) | 232 (77.4) | 42 (13.9) | 259 (86.1) | 56 (18.5) | 245 (81.5) | 15  (4.8) | 286 (95.2) | 117 (38.9) | 183 (61.1) | 39 (13.1) | 261 (86.9) | 5  (1.5) | 296 (98.5) | 25 (8.4) | 275 (91.6) |
| Chittagong | 193 (14.3) | 1,158 (85.7) | 209 (15.5) | 1,141 (84.5) | 173 (12.8) | 1,178 (87.2) | 94  (7.0) | 1,257 (93.0) | 256 (19.0) | 1,095 (81.0) | 166 (12.3) | 1,184 (87.7) | 41  (3.0) | 1,310 (97.0) | 281 (20.8) | 1,069 (79.2) |
| Dhaka | 251 (17.2) | 1,210 (82.8) | 285 (19.5) | 1,176 (80.5) | 274 (18.7) | 1,187 (81.3) | 88  (6.0) | 1,372 (94.0) | 366 (25.1) | 1,094 (74.9) | 130 (8.9) | 1,330 (91.1) | 53  (3.6) | 1,408 (96.4) | 201 (13.7) | 1,260 (86.3) |
| Khulna | 108 (20.2) | 427 (79.8) | 94 (17.5) | 441 (82.5) | 75 (14.0) | 460 (86.0) | 18  (3.3) | 517 (96.7) | 153 (28.5) | 382 (71.5) | 46  (8.6) | 489 (91.4) | 9  (1.7) | 525 (98.3) | 67 (12.6) | 468 (87.4) |
| Mymensingh | 80 (17.4) | 380 (17.4) | 78 (17.0) | 382 (83.0) | 87 (18.9) | 373 (81.1) | 45  (9.7) | 415 (90.3) | 103 (22.4) | 357 (77.6) | 56 (12.2) | 404 (87.8) | 15  (3.2) | 445 (96.8) | 69 (15.1) | 391 (84.9) |
| Rajshahi | 130 (22.2) | 455 (77.8) | 91.4 (15.6) | 493 (84.4) | 95 (16.2) | 490 (83.8) | 39  (6.7) | 545 (93.3) | 217 (37.0) | 368 (63.0) | 55  (9.4) | 529 (90.6) | 7  (1.2) | 577 (98.8) | 60 (10.3) | 524 (89.7) |
| Rangpur | 108 (20.1) | 428 (79.9) | 98 (18.3) | 438 (81.7) | 71 (13.2) | 465 (86.8) | 26 (4.8) | 510 (95.2) | 189 (35.2) | 347 (64.8) | 83 (15.5) | 453 (84.5) | 6  (1.1) | 530 (98.9) | 63 (11.7) | 473 (88.3) |
| Sylhet | 40 (10.0) | 359 (90.0) | 52 (13.1) | 347 (86.9) | 64 (15.9) | 336 (84.1) | 28  (7.1) | 371 (92.9) | 66 (16.5) | 333 (83.5) | 35  (8.7) | 364 (91.3) | 12  (3.1) | 387 (96.9) | 90 (22.5) | 309 (77.5) |
| *p -* value | <0.001 | | 0.055 | | 0.001 | | 0.014 | | <0.001 | | 0.001 | | 0.047 | | <0.001 | |
