# Supplementary material for: Knowledge of Maternal Health Complications: A Critical Analysis Among Pregnant Women in Bangladesh
Source: PLOS Glob Public Health. 2025 Nov 13;5(11):e0005469. doi: 10.1371/journal.pgph.0005469 (PMC12614543; doi:10.1371/journal.pgph.0005469)
Supplement: S2 Table — (DOCX) [file pgph.0005469.s002.docx]

**S2 Table: Detailed multivariable regression analysis showing predictors of specific Knowledge of maternal health complications among currently pregnant women aged 15 – 49 years in Bangladesh, 2016 BMMS [N = 5,625]**

| **Coefficients** | **Severe headache** | | **Blurred vision** | | **High blood pressure** | | **Edema / Pre-Eclampsia** | | **Convulsion /Eclampsia / Unconsciousness** | | **Excess vaginal bleeding** | | **Foul-smelling discharge with severe fever** | | **Jaundice** | |
| --- | --- | --- | --- | --- | --- | --- | --- | --- | --- | --- | --- | --- | --- | --- | --- | --- |
|  | **Coefficient** | ***P* – value** | **Coefficient** | ***P* – value** | **Coefficient** | ***P* – value** | **Coefficient** | ***P* – value** | **Coefficient** | ***P* – value** | **Coefficient** | ***P* – value** | **Coefficient** | ***P* – value** | **Coefficient** | ***P* – value** |
| **Gestational Age** | | | | | | | | | | | | | | | | |
| 1^st^ Trimester | 1.000 | - | 1.000 | - | 1.000 | - | 1.000 | - | 1.000 | - | 1.000 | - | 1.000 | - | 1.000 | - |
| 2^nd^ Trimester | 0.011 | 0.434 | -0.010 | 0.344 | 0.017 | 0.245 | -0.010 | 0.481 | 0.013 | 0.447 | -0.021 | 0.164 | 0.001 | 0.817 | -0.003 | 0.698 |
| 3^rd^ Trimester | 0.009 | 0.548 | 0.010 | 0.443 | 0.061 | <0.001 | -0.006 | 0.673 | -0.011 | 0.551 | -0.031 | 0.047 | -0.003 | 0.624 | 0.001 | 0.952 |
| **Birth Order** | | | | | | | | | | | | | | | | |
| 0 | 1.000 | - | 1.000 | - | 1.000 | - | 1.000 | - | 1.000 | - | 1.000 | - | 1.000 | - | 1.000 | - |
| 1 | -0.013 | 0.450 | 0.006 | 0.646 | 0.004 | 0.796 | -0.001 | 0.950 | 0.077 | <0.001 | 0.035 | 0.095 | -0.003 | 0.648 | -0.016 | 0.141 |
| 2 – 3 | -0.017 | 0.433 | 0.021 | 0.226 | -0.034 | 0.105 | 0.026 | 0.203 | 0.038 | 0.137 | 0.032 | 0.230 | -0.005 | 0.510 | -0.003 | 0.833 |
| 4 + | -0.002 | 0.954 | 0.061 | 0.061 | 0.001 | 0.979 | 0.020 | 0.599 | 0.065 | 0.151 | 0.066 | 0.145 | -0.018 | 0.173 | 0.010 | 0.725 |
| **Age (years)** | | | | | | | | | | | | | | | | |
| <20 | 1.000 | - | 1.000 | - | 1.000 | - | 1.000 | - | 1.000 | - | 1.000 | - | 1.000 | - | 1.000 | - |
| 20 – 24 | 0.045 | 0.008 | -0.005 | 0.705 | 0.062 | <0.001 | 0.004 | 0.789 | 0.059 | 0.005 | 0.029 | 0.070 | -0.001 | 0.921 | 0.007 | 0.564 |
| 25 – 29 | 0.050 | 0.023 | -0.019 | 0.268 | 0.075 | <0.001 | -0.013 | 0.543 | 0.113 | <0.001 | 0.069 | 0.001 | 0.002 | 0.774 | 0.005 | 0.721 |
| 30 + | 0.049 | 0.071 | -0.018 | 0.404 | 0.078 | 0.002 | 0.023 | 0.398 | 0.093 | 0.007 | 0.038 | 0.134 | 0.015 | 0.188 | -0.0001 | 0.996 |
| **Educational Attainment** | | | | | | | | | | | | | | | | |
| No Education | 1.000 | - | 1.000 | - | 1.000 | - | 1.000 | - | 1.000 | - | 1.000 | - | 1.000 | - | 1.000 | - |
| Primary Incomplete | 0.050 | 0.033 | -0.010 | 0.604 | 0.032 | 0.167 | -0.030 | 0.288 | 0.048 | 0.120 | 0.068 | 0.008 | 0.011 | 0.258 | 0.004 | 0.827 |
| Primary Complete | 0.029 | 0.207 | -0.038 | 0.044 | 0.032 | 0.193 | -0.019 | 0.506 | 0.116 | 0.001 | 0.041 | 0.085 | 0.002 | 0785 | -0.020 | 0.313 |
| Secondary Incomplete | 0.075 | 0.001 | 0.028 | 0.127 | 0.062 | 0.005 | -0.021 | 0.428 | 0.125 | <0.001 | 0.073 | 0.002 | 0.003 | 0.674 | -0.003 | 0.854 |
| Secondary completed or Higher | 0.126 | <0.001 | 0.038 | 0.044 | 0.124 | <0.001 | 0.029 | 0.328 | 0.209 | <0.001 | 0.124 | <0.001 | 0.015 | 0.118 | 0.005 | 0.787 |
| **Access to Any Media (Newspaper, Radio, TV)** | | | | | | | | | | | | | | | | |
| At least once a week | 1.000 | - | 1.000 | - | 1.000 | - | 1.000 | - | 1.000 | - | 1.000 | - | 1.000 | - | 1.000 | - |
| Less than once a week | -0.020 | 0.170 | -0.012 | 0.261 | -0.038 | 0.006 | 0.016 | 0.249 | -0.006 | 0.702 | -0.028 | 0.036 | 0.005 | 0.927 | -0.012 | 0.223 |
| **Wealth Quintile** | | | | | | | | | | | | | | | | |
| Lowest | 1.000 | - | 1.000 | - | 1.000 | - | 1.000 | - | 1.000 | - | 1.000 | - | 1.000 | - | 1.000 | - |
| Second | -0.029 | 0.079 | -0.027 | 0.036 | 0.012 | 0.453 | -0.025 | 0.151 | 0.011 | 0.622 | -0.004 | 0.807 | 0.008 | 0.163 | 0.004 | 0.705 |
| Middle | -0.007 | 0.693 | -0.006 | 0.669 | 0.020 | 0.259 | -0.003 | 0.862 | 0.006 | 0.776 | 0.013 | 0.488 | 0.011 | 0.102 | 0.019 | 0.185 |
| Fourth | -0.015 | 0.435 | -0.023 | 0.133 | 0.028 | 0.135 | -0.011 | 0.570 | 0.011 | 0.630 | -0.007 | 0.724 | 0.003 | 0.690 | 0.002 | 0.866 |
| Highest | -0.008 | 0.728 | 0.001 | 0.955 | 0.053 | 0.021 | 0.042 | 0.078 | 0.086 | 0.006 | 0.025 | 0.321 | 0.007 | 0.390 | -0.010 | 0.545 |
| **Place of Residence** | | | | | | | | | | | | | | | | |
| Urban | 1.000 | - | 1.000 | - | 1.000 | - | 1.000 | - | 1.000 | - | 1.000 | - | 1.000 | - | 1.000 | - |
| Rural | -0.021 | 0.141 | -0.013 | 0.265 | -0.029 | 0.047 | -0.003 | 0.802 | 0.018 | 0.349 | -0.013 | 0.383 | -0.001 | 0.831 | -0.010 | 0.330 |
| **Division** | | | | | | | | | | | | | | | | |
| Dhaka | 1.000 | - | 1.000 | - | 1.000 | - | 1.000 | - | 1.000 | - | 1.000 | - | 1.000 | - | 1.000 | - |
| Barisal | 0.088 | 0.002 | -0.028 | 0.184 | 0.068 | 0.012 | 0.013 | 0.647 | 0.043 | 0.211 | -0.112 | <0.001 | -0.009 | 0.355 | -0.006 | 0.721 |
| Chittagong | -0.063 | 0.001 | -0.076 | <0.001 | -0.050 | 0.008 | -0.056 | 0.003 | -0.099 | <0.001 | -0.014 | 0.489 | -0.013 | 0.101 | 0.011 | 0.416 |
| Khulna | 0.024 | 0.326 | 0.003 | 0.863 | 0.003 | 0.939 | 0.037 | 0.136 | 0.019 | 0.521 | -0.054 | 0.019 | -0.012 | 0.223 | -0.010 | 0.494 |
| Mymensingh | -0.090 | 0.000 | -0.084 | <0.001 | -0.039 | 0.069 | -0.070 | 0.001 | 0.082 | 0.006 | 0.021 | 0.406 | -0.013 | 0.179 | -0.029 | 0.036 |
| Rajshahi | 0.041 | 0.090 | 0.003 | 0.841 | 0.028 | 0.224 | 0.031 | 0.222 | -0.048 | 0.087 | -0.041 | 0.068 | -0.027 | 0.001 | 0.011 | 0.513 |
| Rangpur | -0.014 | 0.536 | -0.030 | 0.105 | -0.021 | 0.371 | -0.025 | 0.308 | 0.040 | 0.189 | -0.054 | 0.020 | -0.008 | 0.382 | 0.023 | 0.214 |
| Sylhet | -0.031 | 0.166 | -0.044 | 0.011 | -0.052 | 0.013 | -0.041 | 0.066 | -0.053 | 0.041 | -0.050 | 0.031 | -0.018 | 0.037 | -0.009 | 0.527 |

**S2 Table (continued)**

| **Coefficients** | **Tetanus** | | **Mal-presentation** | | **Prolonged labor** | | **Obstructed labor** | | | **Delayed cord presentation** | | | **Ruptured membrane** | | **Other** | | **Don’t know** | |
| --- | --- | --- | --- | --- | --- | --- | --- | --- | --- | --- | --- | --- | --- | --- | --- | --- | --- | --- |
|  | **Coefficient** | ***P* – value** | **Coefficient** | ***P* – value** | **Coefficient** | ***P* – value** | **Coefficient** | ***P* – value** | | **Coefficient** | | ***P* – value** | **Coefficient** | ***P* – value** | **Coefficient** | ***P* – value** | **Coefficient** | ***P* – value** |
| **Gestational Age** | | | | | | | | | | | | | | | | | | |
| 1^st^ Trimester | 1.000 | - | 1.000 | - | 1.000 | - | 1.000 | - | 1.000 | | | - | 1.000 | - | 1.000 | - | 1.000 | - |
| 2^nd^ Trimester | 0.033 | 0.027 | 0.004 | 0.781 | 0.024 | 0.107 | -0.008 | 0.004 | 0.004 | | | 0.774 | -0.001 | 0.938 | 0.007 | 0.190 | 0.001 | 0.938 |
| 3^rd^ Trimester | -0.001 | 0.624 | 0.031 | 0.040 | -0.010 | 0.491 | -0.022 | 0.007 | 0.007 | | | 0.696 | 0.007 | 0.561 | 0.006 | 0.325 | -0.009 | 0.485 |
| **Birth Order** | | | | | | | | | | | | | | | | | | |
| 0 | 1.000 | - | 1.000 | - | 1.000 | - | 1.000 | - | | 1.000 | | - | 1.000 | - | 1.000 | - | 1.000 | - |
| 1 | 0.003 | 0.847 | 0.023 | 0.216 | 0.007 | 0.683 | 0.006 | 0.213 | | 0.026 | | 0.213 | 0.010 | 0.139 | 0.010 | 0.181 | -0.021 | 0.180 |
| 2 – 3 | 0.008 | 0.687 | 0.029 | 0.228 | -0.014 | 0.537 | 0.020 | 0.087 | | 0.045 | | 0.087 | 0.017 | 0.602 | 0.006 | 0.542 | -0.043 | 0.022 |
| 4 + | 0.048 | 0.207 | 0.024 | 0.535 | -0.029 | 0.440 | 0.031 | 0.085 | | 0.079 | | 0.085 | 0.081 | 0.180 | 0.001 | 0.965 | -0.091 | 0.004 |
| **Age (years)** | | | | | | | | | | | | | | | | | | |
| <20 | 1.000 | - | 1.000 | - | 1.000 | - | 1.000 | - | | 1.000 | | - | 1.000 | - | 1.000 | - | 1.000 | - |
| 20 – 24 | 0.018 | 0.287 | -0.010 | 0.581 | 0.025 | 0.126 | 0.040 | 0.041 | | 0.040 | | 0.041 | 0.019 | 0.139 | -0.001 | 0.891 | -0.079 | <0.001 |
| 25 – 29 | 0.042 | 0.043 | 0.005 | 0.802 | 0.065 | 0.002 | 0.025 | 0.319 | | -0.025 | | 0.319 | 0.008 | 0.602 | -0.001 | 0.828 | -0.091 | <0.001 |
| 30 + | 0.051 | 0.053 | 0.013 | 0.658 | 0.048 | 0.086 | 0.006 | 0.844 | | -0.006 | | 0.844 | 0.032 | 0.180 | 0.009 | 0.432 | -0.089 | <0.001 |
| **Educational Attainment** | | | | | | | | | | | | | | | | | | |
| No Education | 1.000 | - | 1.000 | - | 1.000 | - | 1.000 | - | | 1.000 | | - | 1.000 | - | 1.000 | - | 1.000 | - |
| Primary Incomplete | 0.064 | 0.002 | 0.050 | 0.042 | -0.006 | 0.820 | 0.036 | 0.251 | | 0.036 | | 0.251 | 0.040 | 0.073 | -0.003 | 0.741 | -0.093 | <0.001 |
| Primary Complete | 0.056 | 0.008 | 0.054 | 0.035 | 0.002 | 0.926 | 0.007 | 0.811 | | 0.007 | | 0.811 | 0.046 | 0.048 | -0.005 | 0.616 | -0.056 | 0.039 |
| Secondary Incomplete | 0.112 | <0.001 | 0.025 | 0.251 | 0.014 | 0.575 | -0.018 | 0.536 | | -0.018 | | 0.536 | 0.026 | 0.210 | -0.001 | 0.965 | -0.079 | <0.001 |
| Secondary completed/ Higher | 0.165 | <0.001 | 0.081 | 0.002 | 0.023 | 0.413 | -0.001 | 0.988 | | -0.001 | | 0.988 | 0.036 | 0.123 | 0.013 | 0.341 | -0.143 | <0.001 |
| **Access to Any Media (Newspaper, Radio, TV)** | | | | | | | | | | | | | | | | | | |
| At least once a week | 1.000 | - | 1.000 | - | 1.000 | - | 1.000 | - | 1.000 | | - | | - | - | 1.000 | - | 1.000 | - |
| Less than once a week | 0.002 | 0.834 | -0.007 | 0.606 | -0.033 | 0.015 | -0.015 | 0.059 | -0.014 | | 0.398 | | - | - | 0.001 | 0.894 | 0.005 | 0.721 |
| **Wealth Quintile** | | | | | | | | | | | | | | | | | | |
| Lowest | 1.000 | - | 1.000 | - | 1.000 | - | 1.000 | - | | 1.000 | | - | 1.000 | - | 1.000 | - | 1.000 | - |
| Second | 0.005 | 0.767 | -0.012 | 0.469 | -0.031 | 0.063 | -0.023 | 0.291 | | -0.023 | | 0.291 | -0.002 | 0.838 | -0.014 | 0.032 | -0.003 | 0.838 |
| Middle | -0.016 | 0.387 | 0.029 | 0.133 | -0.028 | 0.112 | -0.014 | 0.217 | | -0.019 | | 0.431 | 0.011 | 0.458 | -0.001 | 0.965 | 0.015 | 0.389 |
| Fourth | -0.004 | 0.816 | 0.004 | 0.832 | -0.027 | 0.156 | -0.015 | 0.076 | | 0.008 | | 0.733 | -0.006 | 0.675 | -0.008 | 0.320 | -0.025 | 0.170 |
| Highest | -0.022 | 0.329 | 0.014 | 0.514 | -0.008 | 0.723 | -0.005 | 0.522 | | 0.007 | | 0.792 | -0.012 | 0.538 | -0.014 | 0.363 | -0.043 | 0.035 |
| **Place of Residence** | | | | | | | | | | | | | | | | | | |
| Urban | 1.000 | - | 1.000 | - | 1.000 | - | 1.000 | - | | 1.000 | | - | 1.000 | - | 1.000 | - | 1.000 | - |
| Rural | 0.023 | 0.089 | 0.001 | 0.906 | 0.013 | 0.358 | 0.004 | 0.023 | | 0.023 | | 0.163 | -0.006 | 0.581 | 0.004 | 0.549 | -0.018 | 0.168 |
| **Division** | | | | | | | | | | | | | | | | | | |
| Dhaka | 1.000 | - | 1.000 | - | 1.000 | - | 1.000 | - | | 1.000 | | - | 1.000 | - | 1.000 | - | 1.000 | - |
| Barisal | 0.039 | 0.173 | -0.051 | 0.035 | -0.011 | 0.653 | -0.016 | 0.257 | | 0.139 | | 0.006 | 0.037 | 0.095 | -0.025 | 0.030 | -0.066 | 0.002 |
| Chittagong | -0.035 | 0.054 | -0.037 | 0.058 | -0.060 | 0.001 | 0.005 | 0.692 | | -0.060 | | 0.006 | 0.030 | 0.045 | -0.007 | 0.449 | 0.066 | <0.001 |
| Khulna | 0.010 | 0.641 | -0.021 | 0.336 | -0.051 | 0.017 | -0.027 | 0.034 | | 0.038 | | 0.153 | -0.005 | 0.723 | -0.022 | 0.033 | -0.011 | 0.580 |
| Mymensingh | -0.002 | 0.898 | -0.021 | 0.333 | 0.001 | 0.980 | 0.032 | 0.059 | | -0.031 | | 0.247 | 0.026 | 0.138 | -0.006 | 0.600 | 0.003 | 0.878 |
| Rajshahi | 0.041 | 0.071 | -0.033 | 0.116 | -0.027 | 0.214 | 0.004 | 0.756 | | 0.126 | | <0.001 | 0.004 | 0.818 | -0.026 | 0.005 | -0.050 | 0.006 |
| Rangpur | 0.013 | 0.600 | -0.010 | 0.681 | -0.062 | 0.005 | -0.016 | 0.276 | | 0.099 | | 0.001 | 0.061 | 0.002 | -0.029 | 0.004 | -0.028 | 0.161 |
| Sylhet | -0.067 | 0.001 | -0.056 | 0.009 | -0.032 | 0.143 | 0.004 | 0.773 | | -0.098 | | <0.001 | -0.008 | 0.657 | 0.005 | 0.652 | 0.081 | 0.001 |
