# Supplementary material for: Knowledge of Maternal Health Complications: A Critical Analysis Among Pregnant Women in Bangladesh
Source: PLOS Glob Public Health. 2025 Nov 13;5(11):e0005469. doi: 10.1371/journal.pgph.0005469 (PMC12614543; doi:10.1371/journal.pgph.0005469)
Supplement: Attachment — Submitted filename: Response to Reviewers Comments_8 October 2025.docx [file pgph.0005469.s003.docx]

October 7, 2025

**RE: PGPH-D-25-01545
Knowledge of Maternal Health Complications: A Critical Analysis Among Pregnant Women in Bangladesh**

**Table 1. Reviewer’s Comments and Author’s Responses**

|  | **Reviewer’s Comments** | **Author’s Response** |
| --- | --- | --- |
| **Reviewer #1** | | |
| **1** | The title suggest it is a 'Critical analysis'; I do not see any justification why the authors think it is a 'critical' analysis. | In this study, we describe our work as a *critical analysis* because it extends beyond reporting prevalence estimates of knowledge of obstetric danger signs. By applying Andersen’s behavioral model, we systematically examine disparities across sociodemographic and pregnancy-related factors, evaluate whether current awareness levels are adequate in light of national and global maternal health goals, and interpret the findings within the context of existing policy and programmatic strategies. This structured and comparative approach allows us to identify systemic gaps and inequities, thereby offering critical insights that can inform targeted interventions and strengthen maternal health outcomes in Bangladesh. |
| **2** | The paper looked at pregnant women's knowledge about maternal complications, which is different from knowledge on obstetric danger signs (ODS). Knowledge of danger signs refers to a pregnant person's awareness of specific warning symptoms (like severe bleeding or reduced fetal movement) whereas knowledge of pregnancy complications is a broader understanding of the serious conditions that can arise during pregnancy, labor, or the postpartum period, such as preeclampsia or gestational diabetes. I suggest to use the term knowledge on maternal complications, not ODS | We thank the reviewer for this valuable clarification. We agree that “knowledge on maternal complications” more accurately reflects the scope of our outcome variable, which encompasses awareness of serious maternal health conditions rather than only specific warning signs. Accordingly, we have revised the terminology throughout the manuscript, replacing “knowledge on obstetric danger signs (ODS)” with “knowledge on maternal complications” to ensure conceptual accuracy and consistency. All corresponding abbreviations, tables, and figure captions have been updated to reflect this change. |
| **3** | Number of birth order (parity) is directly related to the age of the woman; putting both the variables in the model may give spurious results | We thank the reviewer for this valuable point. While age and parity are correlated, our multicollinearity test (VIF = 2.06) indicated no serious concern. We retained both because they capture distinct aspects of maternal experience: age reflects biological and life-stage factors, while parity reflects cumulative exposure to pregnancy and childbirth.  There are several articles where maternal age and parity (birth order) are used together.  Tafazoli, M., Saeedi, R., Gholami Robatsangi, M., & Mazloom, R. (2014). Maternal knowledge on postpartum care in healthcare centers of Mashhad, Iran in 2013. *Journal of Midwifery and Reproductive Health*, 2(4), 207–214. <https://jmrh.mums.ac.ir/?_action=articleInfo&article=4810>  Karkee R, Lee AH, Khanal V. Need factors for utilisation of institutional delivery services in Nepal: an analysis from Nepal Demographic and Health Survey, 2011. BMJ Open 2014;4:e004372. doi:10.1136/bmjopen-2013- 004372 |
| **4** | I suggest to reduce the number of categories of education; no education and primary incomplete could be in one category, primary complete and secondary incomplete could be another category and secondary complete and higher could be another category. This will help to make concrete recommendation on the importance of education. | We appreciate the reviewer’s thoughtful suggestion. We tested the analysis using the proposed condensed education categories and found that the results and overall interpretation remained similar. However, collapsing the categories reduced the ability to capture the nuanced, stepwise gradient of educational attainment observed in our data. To preserve this granularity and better reflect the differential effects of each education level on knowledge of maternal complications, we decided to retain the original five-category classification of educational attainment in the final analysis. |
| **5** | As the relationship of wealth quintiles varied between models, I suggest to group upper two quintiles and lower three quintiles together (40/60), to see whether there is any relationship with the wealth quintiles. | We thank the reviewer for this helpful suggestion. We examined the analysis using the proposed grouping of wealth quintiles (upper two vs. lower three). The results and overall interpretation remained consistent with our primary findings. However, this grouping masked the subtle, nonlinear variations across the five quintiles observed in the original analysis. To retain this level of detail and better illustrate the gradient effect of household wealth on knowledge of maternal complications, we decided to maintain the original five-category classification of wealth quintiles in the final model. |
| **6** | Do you have any other variables such as media exposure or exposure of any other source of information (like club membership), which can explain why some women have some understanding about maternal complications and some others do not? | We thank the reviewer for this insightful comment. We have included **access to any media** (television, radio, or newspapers) as an enabling factor in our analysis to capture women’s exposure to information sources. This variable was found to be a significant predictor of knowledge of maternal complications—women with media access were more likely to recognize key complications such as severe headaches, high blood pressure, and convulsions compared to those without media exposure. Unfortunately, the dataset did not include information on other sources of community or group engagement, such as club membership or participation in women’s organizations. |
| **Reviewer 2** | | |
| **7** | Use of OLS Regression for Binary Outcome: The primary outcome (“sufficient knowledge” ≥3 ODS) is binary, yet OLS regression is used here. This requires justification as logistic regression would typically be preferred. If OLS is retained, you should explain why (e.g., interpretability, comparability) and discuss limitations. | We thank the reviewer for this important observation. We acknowledge that logistic regression is typically recommended for binary outcomes. We opted to use Ordinary Least Squares (OLS) regression because it provides easily interpretable coefficients (mean differences in probability of sufficient knowledge) and facilitates comparability with previous DHS/BMMS-based studies that have employed linear probability models. To ensure robustness, we assessed model assumptions and found no evidence of problematic specification; multicollinearity was also not a concern (mean VIF = 2.06).  We agree that OLS has limitations, including potential issues with heteroskedasticity and predicted values outside the [0,1] range. We will clarify in the methods that OLS was used as a linear probability model for interpretability and comparability, while also acknowledging these limitations in the discussion.  In the manuscript, we have mentioned “A model of the relationship between outcome variables and covariates was developed using multivariable Ordinary Least Squares (OLS) regressions employing a linear probability model for interpretability and comparability to calculate coefficients at 95% confidence intervals (CIs).”, in page 8, lines 169 - 171. |
| **8** | Definition of Outcome Variable: The threshold of ≥3 signs to define “sufficient knowledge” appears subjective. Please provide citations or theoretical justification for this cut-off. Without justification, readers may question the validity of the outcome. | We appreciate the reviewer’s important observation. To address this, we have added justification in the Methods section in Page 8, lines 148 – 155, “Considering prior studies …………. as emphasized in WHO guidance [23].” |
| **9** | Results: For multivariable models, the manuscript currently lists β coefficients without interpretation. It would be good to translate some findings into percentage point differences or practical meaning such as “Women with higher education were xx percentage points more likely to recognize ≥3 signs compared to those with no education”. | We thank the reviewer for this suggestion. We have revised the Results section to provide a clearer interpretation of key β coefficients in terms of percentage point differences. For example, we now state that *“Women with completed secondary or higher education were nearly 18 percentage points more likely to recognize three or more obstetric danger signs compared to women with no education”.* These additions help translate the regression findings into practical, policy-relevant terms. |
| **10** | Model Presentation: The description of Models 1 to 3 is not well-integrated into the results narrative. Currently it is unclear how the models build on each other. Kindly summarize briefly what each model adds and how key predictors change across models. | We thank the reviewer for this helpful comment. In response, we revised the Results section on pages 13-16, lines 240-298, to more clearly describe how Models 1–3 build on one another and how the inclusion of covariates affects the predictors. We also integrated coefficients and p-values from Table 3 directly into the narrative to highlight how associations change across models. Additionally, we condensed the detailed regression findings (from S2 Table). These revisions ensure that the models are now presented as a coherent sequence, with a clearer interpretation of how results evolve across models. |
| **11** | Regional Disparities: The results highlight regional differences (e.g., Sylhet vs. Dhaka) but the interpretation is limited. A stronger discussion of why these disparities exist (sociocultural, health system, service access) would strengthen the manuscript. | We thank the reviewer for this helpful suggestion. We agree that the discussion of regional disparities can be strengthened. In the revised manuscript, we will expand our interpretation to note that regions such as “Sylhet and Chittagong have historically lagged behind Dhaka due to a combination of sociocultural norms (e.g., greater restrictions on women’s mobility and decision-making), geographic and infrastructural challenges (hard-to-reach areas with limited transport), and weaker health system readiness and outreach compared to central divisions.” These factors have been documented in prior evaluations of the Health, Population and Nutrition Sector Program (HPNSP) and help explain why women in Dhaka demonstrate higher knowledge than those in Sylhet. We will integrate these contextual explanations to provide a more nuanced interpretation of the observed disparities., in Page 18, lines 316 – 319. |
| **12** | Terminology: Inconsistent use of terms (“pregnancy complications” vs. “obstetric danger signs”). Please choose one and use consistently throughout. | We appreciate the reviewer’s observation. To ensure conceptual clarity and consistency, we have standardized the terminology throughout the manuscript. As suggested, we now consistently use the term **“**maternal complications**”** instead of “obstetric danger signs,” since it more accurately represents the broader scope of women’s knowledge assessed in this study. All sections, including the Abstract, Methods, Results, Discussion, tables, and figure captions, have been revised accordingly. |
