# Supplementary material for: Knowledge of Maternal Health Complications: A Critical Analysis Among Pregnant Women in Bangladesh
Source: PLOS Glob Public Health. 2025 Nov 13;5(11):e0005469. doi: 10.1371/journal.pgph.0005469 (PMC12614543; doi:10.1371/journal.pgph.0005469)
Supplement: Attachment — Submitted filename: Response to Reviewers_15 October 2025.docx [file pgph.0005469.s004.docx]

October 15, 2025

**RE: PGPH-D-25-01545
Knowledge of Maternal Health Complications: A Critical Analysis Among Pregnant Women in Bangladesh**

**Journal Requirements:**

If the reviewer comments include a recommendation to cite specific previously published works, please review and evaluate these publications to determine whether they are relevant and should be cited. There is no requirement to cite these works unless the editor has indicated otherwise. 

Please review your reference list to ensure that it is complete and correct. If you have cited papers that have been retracted, please include the rationale for doing so in the manuscript text, or remove these references and replace them with relevant current references. Any changes to the reference list should be mentioned in the rebuttal letter that accompanies your revised manuscript. If you need to cite a retracted article, indicate the article’s retracted status in the References list and also include a citation and full reference for the retraction notice.

Additional Editor Comments (if provided):

[Note: HTML markup is below. Please do not edit.]

Answer: We would like to thank the Journal Editor for this comment. The title of Fig 1 has been revised as “Fig 1: Conceptual framework of factors associated with knowledge of maternal complications and health care-seeking behavior in Bangladesh” on Page 7, lines 128-129.

The reference [17] will not be deleted, as the conceptual framework presented in this study is based on reference 17, which we mentioned on Page 6, line 114.

In this revised version, we have added two references, mentioned as numbers 22 and 23 in the title “References” on page 8, lines 158 and 162. The added references are:

22. Lahole, B.K., Mare, K.U., Shewangizaw, M. *et al.* Analyzing women’s knowledge of pregnancy complications in Ethiopia through a multilevel approach. *Sci Rep* **14**, 24332 (2024). <https://doi.org/10.1038/s41598-024-75152-3>.

23. World Health Organization (WHO). *Pregnancy, childbirth, postpartum and newborn care: A guide for essential practice (3rd edition).* Geneva: WHO; 2015. Available from: <https://www.who.int/publications/i/item/9789241549356>.

We have deleted the following reference as it was duplicated.

25. Bolanko A, Namo H, Minsamo K, Addisu N, Gebre M. Knowledge of obstetric danger signs and associated factors among pregnant women in Wolaita Sodo town, South Ethiopia: A community-based cross-sectional study. SAGE Open Med. 2021; 9:20503121211001161.

**Reviewers' comments:**
[NOTE: If reviewer comments were submitted as an attachment file, they will be attached to this email and accessible via the submission site. Please log into your account, locate the manuscript record, and check for the action link "View Attachments". If this link does not appear, there are no attachment files.]

**Figure Resubmissions:**
While revising your submission, we strongly recommend that you use PLOS’s NAAS tool (https://ngplosjournals.pagemajik.ai/artanalysis) to test your figure files. NAAS can convert your figure files to the TIFF file type and meet basic requirements (such as print size, resolution), or provide you with a report on issues that do not meet our requirements and that NAAS cannot fix.

After uploading your figures to PLOS’s NAAS tool - https://ngplosjournals.pagemajik.ai/artanalysis, NAAS will process the files provided and display the results in the "Uploaded Files" section of the page as the processing is complete. If the uploaded figures meet our requirements (or NAAS is able to fix the files to meet our requirements), the figure will be marked as "fixed" above. If NAAS is unable to fix the files, a red "failed" label will appear above. When NAAS has confirmed that the figure files meet our requirements, please download the file via the download option, and include these NAAS processed figure files when submitting your revised manuscript.

Answer: We would like to thank to the editor for bringing up this issue. We have uploaded both Fig 1 and Fig 2, using PLOS’s NAAS (<https://ngplosjournals.pagemajik.ai/artanalysis>) according to the PLOS GPH Journal requirements.
