# Supplementary material for: Knowledge of Maternal Health Complications: A Critical Analysis Among Pregnant Women in Bangladesh
Source: PLOS Glob Public Health. 2025 Nov 13;5(11):e0005469. doi: 10.1371/journal.pgph.0005469 (PMC12614543; doi:10.1371/journal.pgph.0005469)
Supplement: Attachment — Submitted filename: Response to Reviewers_21 October 2025.docx [file pgph.0005469.s005.docx]

October 21, 2025

**RE: PGPH-D-25-01545
Knowledge of Maternal Health Complications: A Critical Analysis Among Pregnant Women in Bangladesh**

**Journal Requirements:**

1. Please provide a detailed online Financial Disclosure statement. This is published with the article. It must therefore be completed in full sentences and contain the exact wording you wish to be published.

a) Please clarify all sources of financial support for your study. List the grants, grant numbers, and organizations that funded your study, including funding received from your institution. Please note that suppliers of material support, including research materials, should be recognized in the Acknowledgements section rather than in the Financial Disclosure. 
b) State the initials, alongside each funding source, of each author to receive each grant. For example: “This work was supported by the National Institutes of Health (####### to AM; ###### to CJ) and the National Science Foundation (###### to AM).”
c) State what role the funders took in the study. If the funders had no role in your study, please state: “The funders had no role in study design, data collection and analysis, decision to publish, or preparation of the manuscript.”

For more information, please go to our submission guidelines:
<https://journals.plos.org/globalpublichealth/s/submission-guidelines#loc-financial-disclosure-statement>

We would like to thank the editor for this review comment. We updated the Financial Disclosure statement according to the PLOS submission guidelines as “**Financial Disclosure:** This work was supported by the Carolina Population Center and its National Institutes of Health (NIH) Center for Scientific Review Grant (#R24 HD050924) and by the United States Agency for International Development (USAID) through the Data for Impact (D4I) Associate Award number 7200AA18LA00008. The funders had no role in study design, data collection and analysis, decision to publish, or preparation of the manuscript.”

2. Please ensure that the funders and grant numbers match between the Financial Disclosure field and the Funding Information tab in your submission form. Note that the funders must be provided in the same order in both places as well.

We are ensuring that the funders and grant numbers are matched between the Financial Disclosure field and the Funding Information tab in our submission form, provided in the same order in both places.

Here, we mentioned this section as “Funder: United States Agency for International Development (USAID); Grant: National Institutes of Health (NIH) Center for Scientific Review Grant number R24 HD050924.”

3. Please update your online Competing Interests statement. If you have no competing interests to declare, please state: “The authors have declared that no competing interests exist.”

This is to inform the editor that we have updated our online Competing Interests statement as “The authors have declared that no competing interests exists.”

4. In this instance it seems there may be acceptable restrictions in place that prevent the public sharing of your minimal data. However, in line with our goal of ensuring long-term data availability to all interested researchers, PLOS’ Data Policy states that authors cannot be the sole named individuals responsible for ensuring data access (<http://journals.plos.org/globalpublichealth/s/data-availability#loc-acceptable-data-sharing-methods>).

Data requests to a non-author institutional point of contact, such as a data access or ethics committee, helps guarantee long term stability and availability of data. Providing interested researchers with a durable point of contact ensures data will be accessible even if an author changes email addresses, institutions, or becomes unavailable to answer requests.

Before we proceed with your manuscript, please also provide non-author contact information (phone/email/hyperlink) for a data access committee, ethics committee, or other institutional body to which data requests may be sent. If no institutional body is available to respond to requests for your minimal data, please consider if there any institutional representatives who did not collaborate in the study, and are not listed as authors on the manuscript, who would be able to hold the data and respond to external requests for data access? If so, please provide their contact information (i.e., email address). Please also provide details on how you will ensure persistent or long-term data storage and availability.

We would like to thank to the editor for bringing up this issue. We have updated the ‘Data Availability Statement’ as “This study This study analyzed publicly available, de-identified data from the 2016 Bangladesh Maternal Mortality and Health Care Survey (BMMS), conducted by the NIPORT, icddr,b, and MEASURE Evaluation. The dataset can be accesses through the UNC Dataverse at <https://doi.org/10.15139/S3/X33NIZ> to all qualified researchers.

For long-term and stable data access, requests for additional details about the BMMS dataset or related metadata may be directed to the Data Access Committee, Carolina Population Center (CPC), University of North Carolina at Chapel Hill (email: [dataverse@unc.edu](mailto:dataverse@unc.edu); Phone: +1-919-962-3000).” In Page 22, Lines 394 - 400.

5. We have noticed that you have uploaded Supporting Information files, but you have not included a list of legends. Please add a full list of legends for your Supporting Information files before or after the references list.

We would like to thank to the editor for this comment. We have included a list of legends for our Supporting Information files before the reference list, in Page 23, Lines 413 - 420.

6. If the reviewer comments include a recommendation to cite specific previously published works, please review and evaluate these publications to determine whether they are relevant and should be cited. There is no requirement to cite these works unless the editor has indicated otherwise.

We would like to thank to the editor for this comment. We have checked all the in-word citations and references incorporated in this manuscript.

7. Please review your reference list to ensure that it is complete and correct. If you have cited papers that have been retracted, please include the rationale for doing so in the manuscript text, or remove these references and replace them with relevant current references. Any changes to the reference list should be mentioned in the rebuttal letter that accompanies your revised manuscript. If you need to cite a retracted article, indicate the article’s retracted status in the References list and also include a citation and full reference for the retraction notice.

Answer: We would like to thank the Journal Editor for this comment. We are ensuring that the reference list is complete and correct. We have not cited any papers that have been retracted.

Additional Editor Comments (if provided):

No Additional Editor Comments.


[Note: HTML markup is below. Please do not edit.]

Reviewers' comments:

No Reviewer’s comments.

[NOTE: If reviewer comments were submitted as an attachment file, they will be attached to this email and accessible via the submission site. Please log into your account, locate the manuscript record, and check for the action link "View Attachments". If this link does not appear, there are no attachment files.]

Figure Resubmissions:

While revising your submission, we strongly recommend that you use PLOS’s NAAS tool (<https://ngplosjournals.pagemajik.ai/artanalysis>) to test your figure files. NAAS can convert your figure files to the TIFF file type and meet basic requirements (such as print size, resolution), or provide you with a report on issues that do not meet our requirements and that NAAS cannot fix.

After uploading your figures to PLOS’s NAAS tool - <https://ngplosjournals.pagemajik.ai/artanalysis>, NAAS will process the files provided and display the results in the "Uploaded Files" section of the page as the processing is complete. If the uploaded figures meet our requirements (or NAAS is able to fix the files to meet our requirements), the figure will be marked as "fixed" above. If NAAS is unable to fix the files, a red "failed" label will appear above. When NAAS has confirmed that the figure files meet our requirements, please download the file via the download option, and include these NAAS processed figure files when submitting your revised manuscript.

We would like to thank to the editor for bringing up this issue. We have uploaded both Fig 1 and Fig 2, using PLOS’s NAAS (<https://ngplosjournals.pagemajik.ai/artanalysis>) according to the PLOS GPH Journal requirements.
